# Incidence, risk factors and outcomes of traumatic head injury among trauma patients visited at the Yanet Trauma and Surgery Specialized Centre, Sidama region, Hawassa, Ethiopia: cohort study

**DOI:** 10.3389/fneur.2024.1431999

**Published:** 2024-09-30

**Authors:** Tadelech Abebe, Tsegaye Alemu, Mende Mensa Sorato

**Affiliations:** ^1^Department of Public Health, Yanet-Liyana College of Health Sciences, Hawassa, Ethiopia; ^2^School of Public Health, College of Medicine and Health Sciences, Hawassa University, Hawassa, Ethiopia; ^3^Department of Pharmacy, School of Medicine, Komar University of Science and Technology, Sulaymaniyah, Iraq

**Keywords:** head injury, incidence, risk factor, trauma, Sidama Ethiopia

## Abstract

**Introduction:**

Traumatic brain injuries are a major public health concern that contributes to youth morbidity and mortality in developing nations, including Ethiopia. Despite of this, little is known about head injury in the study area. The goal of the study was to identify the incidence, risk factors and outcomes for traumatic head injury among trauma patients who visited at the Yanet Trauma and Surgery Specialized Centre.

**Methods:**

This was a 5 year an institutional-based retrospective cohort study conducted among 1,029 patients who experienced trauma and admitted at the Yanet Trauma and Surgical Specialized Centre. The research was carried out between September 01/2023 to October 15/2023. The study units were selected by using simple random sampling techniques through computer-generated random numbers. The data were collected via a checklist designed on the Kobo toolbox with a smart smartphone. The collected data were exported to a statistical package for Social Science version 27. Then, descriptive statistical analysis was conducted to determine the mean, standard deviation, and median. Bivariate and multivariate logistic regression was subsequently conducted to determine the associations between head injury and the independent variables.

**Result:**

A total of 1,029 injured patients were followed for 2,302 person-days. Over all, incidence density rate of 14.03/100 person-days (323, 31.4%) [95% CI: 29.5–34%]. The third year of follow-up showed the greatest incidence compared to other years. The most common type of head injury observed during following up were brain contusion (38.1%), followed by epidural hematoma (33.1%), skull fracture (15.8%), and intracerebral hematoma (13.0%). In multivariate logistic model, rural residence [AOR = 1.6; 95% CI: 1.18–2.16], mechanism of injury namely road traffic accident [AOR = 5.5; 95% CI: 2.27–13.34], assault [AOR = 3.4; 95% CI: 1.35–8.37] and comorbidity of chronic disease [AOR = 2.2; 95% CI: 1.13–4.18] were the risk factors significantly associated with head injury.

**Discussions:**

The incidence density rate of 14.03/100 person-days. As the result, more has to be done by health professionals, traffic police officers and local government raise awareness and enforce the implementation of driving rules and regulations.

## 1 Introduction

Head injury, often known as traumatic brain injury (TBI), refers to damage to the brain caused by rapid trauma. The extent of the injury might vary, depending on which parts of the brain are damaged (1). Worldwide, the occurrence of TBI is estimated to range from 27 to 69 million cases per year, with an average of 801 incidents per 100,000 patients annually, according to the WHO (2–4). In low-and middle-income countries (LMICs), the burden of injury is high, particularly among where more than 90% of deaths are due to injuries (8).

The WHO states that traumatic head injury is a major public health concern (5) due to its frequent occurrence as a cause of death and disability among young individuals, and its substantial impact on health care system (6). Approximately, 5.3 million individuals in the United States and 7.7 million individuals in the European Union are estimated to have impairments resulting from traumatic brain injury (7, 8). Head injuries are the main cause of disability and death worldwide and have become one of the largest public health challenges, including in Ethiopia (9, 10). The present research reveals that it is the leading cause of death, especially in low-to middle-income countries (11).

According to epidemiological research, about 57 million individuals globally are experiencing the neurological effects of severe head traumas (11, 12). Nevertheless, a significant number of calculations relied on data from industrialized nations, which lacked sufficient representation of low-and middle-income countries, especially in the African area, such as Ethiopia. Besides, untreated intracranial injuries, such as epidural and subdural hemorrhages or skull fractures, may lead to mortality or persistent impairment (13, 14). Therefore, Traumatic Brain Injury (TBI) poses a substantial worldwide risk to public health, affecting about 69 million people annually on a global scale (3, 15). Moreover, a significant percentage of traumatic brain injuries were recorded in low-income countries (3). Furthermore, road traffic accidents (RTAs), fall-down accidents, and interpersonal violence (assaults) were the major risk factors for head injury (16, 17). Therefore, the World Assembly considered RTAs to be one of the agenda for Sustainable Development Goals (18).

Moreover, the Yanet Trauma and Surgery Specialized Center is the largest trauma center in Hawassa, serving more than 4 regions in Ethiopia (patients from the Oromia, Southern, Sidama, and Somalia regions). However, no studies have investigated the incidence, risk factors, and outcomes of traumatic head injury among trauma patients in the study area.

Therefore, this study aimed to determine incidence, risk factors and outcomes for traumatic head injury among trauma patients who visited at the Yanet Trauma and Surgery Specialized Centre.

## 2 Methods and materials

### 2.1 Study design

An institution-based retrospective cohort study was conducted.

### 2.2 Study area and period

This study was conducted at Yanet Trauma and Surgery Specialized Center, Hawassa, Ethiopia. The Yanet Trauma and Surgery Specialized Center (YTSSC) is a tertiary health institution designed to provide services to two specialized centers under one roof, namely, the Yanet Trauma Specialized Center and the Yanet Surgery Specialized Center. It is a private health institution under the Liyana Health Care PLC; it was inaugurated and started functioning on January 12, 2019. Tertiary care is provided in both diagnostic and therapeutic services. As it is the only specialized trauma and surgery center in the Sidama region, its service is also focused especially on trauma. It offers diagnosis and treatment services for an average patient flow of 13,473 patients, which includes 2026 trauma patients and 11,447 non-trauma patients per year since 2014. The study was conducted from September 01/2023 to October 15/2023.

### 2.3 Population

#### 2.3.1 Source population

The source population included all trauma patients who visited the YTSSC from July 2019 to July 2023.”

#### 2.3.2 Study population

All randomly selected trauma patients charts at the YTSSC from July 2019 to July 2023.

### 2.4 Inclusion and exclusion criteria

#### 2.4.1 Inclusion criteria

All trauma patient cards registered at the Yanet Trauma and Surgery Specialized Centre from July 2019 to July 2023 were eligible for the study.

#### 2.4.2 Exclusion criteria

Patient records with incomplete information were excluded from the study.

### 2.5 Sample size determination

#### 2.5.1 Sample size for the first objective

In this study, we considered a single and double population proportion formula. Using a 95% confidence interval, 3% margin of error, and 40.5% incidence of head injury from a previous study (19), the calculated sample size was 1,029.

#### 2.5.2 Sample size for the second objective

The double population formula was used for sample size determination in the second objective. Epi-info version 7.0 with a confidence level of 95% and a power of 80%, and the ratio of the unexposed group to the exposed group was considered to be one. As shown in below assessing the exposure of gender or being male gender requires the highest sample size. Therefore, the final sample size required for conducting this study was 1,029 (19, 20).

### 2.6 Sampling technique and procedure

This study was conducted at Yanet Trauma and Surgery Specialty Center, Sidama region, Hawassa, Ethiopia. A simple random sampling technique was used to select the patients’ charts. Hence, the sampling frame was a registry of trauma patient data registered in Excel from July 2019 to July 2023. Finally, computer-generated random numbers were used to enroll all the study units (*n* = 1,029) from the Excel registry of total trauma patients (*n* = 8,820) who visited the Yanet Trauma and Surgery Specialized Center from July 2019 to July 2023.

### 2.7 Variables

#### 2.7.1 Dependent variables

- Traumatic head injury

#### 2.7.2 Independent variables

Socio-demographic Factors: Age, Sex, Residence.

Mechanism of injury: RTA, fall down accident, Burn, Fall down object, Assault (Fighting, bullet injury). Clinical characteristics: Epilepsy, psychiatric illness, DM, HTN.

### 2.8 Data collection tool and procedures

#### 2.8.1 Data collection tool and data collection procedures

The data collection tool was adapted from the WHO on injury surveillance (21) and the literature, modified to fit the study context after pretesting at Hawassa referral hospital, which was not included in the present study. The questionnaires consisted of five sections, including socio-demographic variables, physical factors, personal factors, RTAs, and clinical factors.

#### 2.8.2 Data collection procedures

Before data collection was carried out, the final version of the structured tool was designed by using the Kobo collection toolbox. Then, two trained professional nurses were recruited as data collectors. These two nurses extracted data from the registry system by randomly selected patients who had a follow-up from 2019 to 2023 under the supervision of the data manager and hospital administrators who were trained for this purpose. The researchers rechecked if there was an incomplete and inconsistent abstraction from the chart every day; if an incomplete checklist was found, we sent it back to the data collectors for correction.

### 2.9 Data quality assurance

To maintain the quality of the data, a pretest was performed on 35 trauma patients at the emergency unit of Hawassa University Comprehensive Specialized Hospital. After the pretest, some revisions were made to the questionnaires, and modifications were made to the skipping pattern. Finally, the pretested Kobo toolbox was used to collect the data. One day of training was given to the data collectors and supervisors. Furthermore, the principal investigator and supervisors supervised the daily process of data collection and provided necessary corrective measures.

### 2.10 Data processing and analysis

The gathered information was downloaded as Excel and SPSS labels, and it was then exported to SPSS version 27 for analysis. For continuous variables, descriptive statistics such as means and standard deviations were computed; for categorical variables, tables and graphs show frequencies and percentages. Binary logistic regression analysis was used to identify risk variables for head injuries. Therefore, factors in the bivariate analysis with *p-values* less than 0.25 were deemed potential candidates for multivariate analysis. Authors also considered certain criteria for the multivariate analysis, took adequate sample size for the analysis, as we know that inadequate sample sizes may lead to unreliable results, especially in multivariate analyses with multiple predictors. We verified the variables were presented and sufficient completed for each observation. Besides, authors checked outliers, normality, and *p-value < 0.25* and also checked multicollinearity among predictor.

In addition, the multivariate binary logistic regression model included a backward likelihood ratio to control for confounding variables. A head injury was deemed to be predicted if the *p- value* was less than 0.05 at the 95% confidence range. Furthermore, the Hosmer and Lemeshow test was used to assess the model’s fitness; a *P- value* of more than 0.05 was seen as indicating a good model fit. Additionally, multicollinearity was tested using the variance inflation factor (VIF) had less than 10.

### 2.11 Operational definitions

Head injury was defined as patients who experienced traumatic scalp and/or skull injuries with or without traumatic brain injury (22). It is refer to a change in brain function that may cause convulsion, coma, and altered level of consciousness called traumatic brain injury (22).

Conservative management: a patient who experienced traumatic scalp or skull injury with or without traumatic brain injury and was treated without any surgical procedure.

## 3 Results

### 3.1 **Socio-demographic** characteristics

In our study, we included 1,029 research respondents for the final analysis. The mean age of the respondents was 35.82 ± 18.53 years. The majority (70.9%, *n* = 730) of the study participants were male. Furthermore, the majority (72.6%, *n* = 747) of the participants were urban residents. Considering the referral history of more than half of the study participants, 55.3% (*n* = 564) had a referral history, and the majority (73.5%, *n* = 338) of the study respondents were referred from governmental health institutions (Table 1).

**Table 1:** Admission characteristics of study participants who visited the Yanet Trauma and Surgery Specialized Center from July 2019 to July 2023.

Socio-demographic variables	Category	Frequency (*n*)	Percent (%)
Sex	Male	730	70.9
	Female	299	29.1
Age	<15 Years	104	10.1
	15–30 Years	381	37.0
	31–45 Years	280	25.3
	> = 45 Years	284	27.6
Residence	Rural	282	27.4
	Urban	747	72.6
Referral history	Yes	460	44.7
	No	569	55.3
Source of referral	Private health institutions	122	26.5
	Public health institutions	338	73.5
**Admission characteristics**			
Mechanism of trauma	Road traffic accident (RTA)	382	37.1
	Fall down accident	396	38.5
	Assault	206	20.0
	Other (Machine injury, animal bite or burn)	45	4.4
Glasgow coma scale at admission	<8	37	3.6
	9–12	115	11.2
	13–15	877	85.2
Blood pressure	Normal	822	79.9
	Hypotension	14	1.4
	Hypertension	193	18.8
Body temperature	Normal	1,001	97.3
	Hypothermia	9	0.9
	Hyperthermia	19	1.8
Pulse rate	Normal	873	84.8
	Bradycardia	54	5.2
	Tachycardia	102	9.9

### 3.2 Admission characteristics of the study participants

The majority of traumas were occurred due to fall-down accidents (38.5%) and road traffic accidents (37.1%). About (85.2%, *n* = 877) of the respondents were presented with Glasgow Coma Scale (GCS) scores ranging from 13 to 15 at admission. Furthermore, the greater proportion of respondents presented with normal vital signs, with percentages of 79.9% (*n* = 822), 84.8% (*n* = 873), and 97.3% (*n* = 1,001) for blood pressure, pulse, and temperature, respectively (Table 1).

### 3.3 Clinical conditions of the patients

Regarding clinical condition, about 58.8% (*n* = 605) of the research respondents were outpatients. In addition, 13.75% (*n* = 141) of the study participants presented with poly trauma, and more than half (58.2%, *n* = 82) of them injured their extremities. In addition, almost one-third (32.9%, *n* = 339) of the study participants presented with bone fractures. Medical comorbidities and visceral organ injury were present in 4.3% (*n* = 44) and 4.7% (*n* = 48) of the study participants, respectively. Among the investigated modalities, the most common were X-ray (58.2%, *n* = 599), followed by head computed tomography (CT) (52.2%, *n* = 537) and electrocardiogram (ECG) (13.4%, *n* = 138) (Table 2).

**Table 2:** Clinical characteristics of the study participants who visited the Yanet Trauma and Surgery Specialized Center from July 2019 to July 2023.

Clinical characteristics	Category	Frequency (*n*)	Percent (%)
Patient treatment condition	Admitted	348	33.8
	Outpatient	605	58.8
	Referred	76	7.4
Skull X-ray	Yes	59	5.7
	No	970	94.3
Head computed tomography (CT)-Scan	Yes	537	52.2
	No	492	47.8
Magnetic resonance imaging (MRI)	Yes	96	9.3
	No	933	90.7
X-ray for other body part	Yes	599	58.2
	No	430	41.8
Electro cardio grapy (ECG)	Yes	138	13.4
	No	891	86.6
Ultrasound (US)	Yes	54	5.2
	No	975	94.8
Poly trauma	Yes	141	13.7
	No	888	86.3
Injured part of poly trauma	Extremity	82	58.2
	Abdomen	10	7.1
	Chest	29	20.8
	Other*	20	14.22
Bone fracture	Yes	339	32.9
	No	690	67.1
Injury to visceral organ	Yes	48	4.7
	No	981	95.3
Injured visceral organ	Spleen	4	8.3
	Liver	3	6.3
	Heart	3	6.3
	Lung	32	66.7
	Genitourinary	6	12.5
Presence of comorbidity	Yes	44	4.3
	No	985	95.7
Type of comorbidity	Diabetes mellitus	10	22.7
	Hypertension	22	50.0
	Epilepsy	1	2.3
	Psychiatric illness	2	4.5
	Tuberculosis (TB)	1	2.3
	Peptic ulcer disease (PUD)	2	4.5
	Osteoarthritis	3	6.8
	Cardiac disease	1	2.3
	Chronic obstructed pulmonary disease (COPD)	2	4.5

*Face, nose, neck, shoulder, zygomatic fracture.

### 3.4 Incidence of head injury

**Figure 1:**
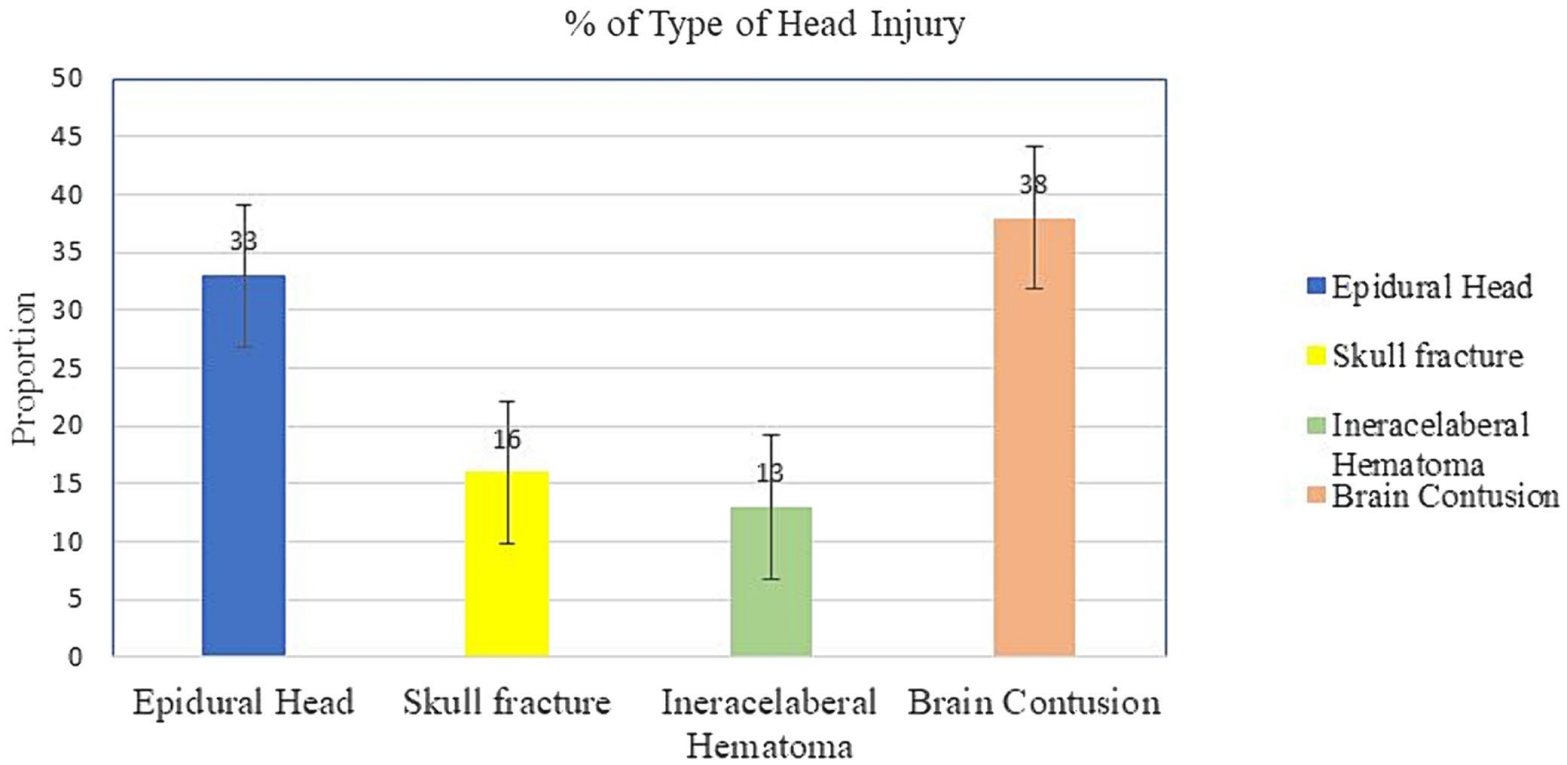
Type of head injury among study respondents from trauma patients who visited the Yanet Trauma and Surgery Specialized Center from July 2019 to July 2023.

In the 5 years, 1,029 injured patient followed 2,302 person-days. The incidence density rate of head injury was 14.03/100 person-days. The cumulative incidence of head injury among trauma patients was 31.4% [95% CI (29.5–34%)].The most common type of head injury was brain contusion (38.1%, *n* = 123), followed by epidural hematoma (EDH) (33.1%, *n* = 107), skull fracture (15.8%, *n* = 51) and intracerebral hematoma (ICH) (13.0%, *n* = 42). Furthermore, only 5.6% (*n* = 57) and 1.7% (*n* = 18) of the study participants developed trauma-related complications and hospital-acquired infections, respectively (Figure 1).

### 3.5 Head injury complication and management

Overall, 15 (5.5%) of the patients developed posttraumatic seizures, 13 (1.3%) developed aspiration pneumonia, 7 (0.7%) developed skin contusions, 7 (0.7%) developed extremity paralysis, 5 (0.5%) developed post-traumatic meningitis, and 4 (0.4%) underwent amputation. Regarding management, 180 (55.7%) of the patients were managed with medication (conservative management), and 143 (44.3%) of the head-injured patients were managed with surgery (Table 3).

**Table 3:** Incidence, management, and trauma-related complications of head injury among study participants from trauma patients who visited the Yanet Trauma and Surgery Specialized Center from July 2019 to July 2023.

Variable	Category	Frequency (n)	Percent (%)
Presence of head injury	Yes	323	31.4
	No	706	68.6
Management given for head injury	Conservative	180	55.7
	Surgical	143	44.3
Trauma-related complications	Yes	57	5.6
	No	956	94.4
	Urinary retention	3	5.3
	Skin contracture	7	12.3
	Amputation	4	7.0
	Aspiration pneumonia	13	22.8
	Post trauma meningitis	5	8.8
	Posttraumatic seizure	15	26.3
	Extremity paralysis	7	12.3
	Decreased hearing	3	5.3
Hospital-acquired infection	Yes	18	1.7
	No	1,011	98.3
Type of hospital-acquired infection	Pneumonia	6	35.3
	Wound infection	11	64.7
Length of hospital stay	Less than or equal to 7 days	269	83.3
	More than 7 days	54	16.7

### 3.6 Head injury outcome

Among the total admitted patients, almost one-quarter of the respondents (242, 23.3%) were cured and returned home, 59 (5.7%) were referred to higher facilities due to complications, 5 (0.5%) had no improvement and reported no improvement, and the remaining 17 (1.7%) of the admitted patients died. The mean (±SD) number of patients who stayed in the trauma center was 4.46 (± 5.11), and the maximum number of stay days was 56 (Table 3).

### 3.7 Predictors of head injury

According to the bivariate analysis, seven variables were significantly associated with head injury at a significance level less than 0.25. Hence, sex, residence, place of trauma, fall-down accident, road traffic accident, assault, and comorbidity were the seven independent candidate variables for multivariable analysis.

After adjusting for confounders through the backward stepwise likelihood ratio in multivariate binary logistic regression, four variables showed a statistically significant association with head injury among trauma patients, namely, residence, RTA, assault, and comorbidity.

The odds of head injury were almost 2 times greater among rural residents than among urban residents (AOR = 1.6; 95% CI: 1.18–2.16). Furthermore, the odds of head injury were almost 6 times greater for road traffic accidents than for other means of injury (AOR = 5.5; 95% CI: 2.27–13.34). Similarly, the odds of head injury were 3.4 times greater for the assault mode than for the other means of injury (AOR = 3.4; 95% CI: 1.35–8.37). Finally, the odds of head injury were 2.2 times greater among patients with comorbidities of chronic disease than among their counterparts (AOR = 2.2; 95% CI: 1.13–4.18) (Table 4).

**Table 4:** Factors associated with head injury among study participants from trauma patients who visited the Yanet Trauma and Surgery Specialized Center from July 2019 to July 2023.

Variable	Head injury		COR [95%CI]	AOR [95%CI]	*p* value
	Yes	No			
**Sex**					
Female	78	221	1	1	
Male	245	485	1.5 [1.06–1.93]	1.3 [0 0.92–1.73]	0.149
**Residence**					
Urban	210	537	1	1	
Rural	113	168	1.7[1.28–2.27]	1.6[1.18–2.16]	0.002*
**Place of trauma**					
Recreational area	9	43	1	1	
Home	51	198	1.2 [0.56–2.69]	1.1 [0 0.50–2.51]	0.779
Workplace	68	208	1.5 [0 0.72–3.37]	1.2 [0 0.56–2.75]	0.579
Street	195	257	3.6[1.72–7.62]	1.6 [0.71–3.52]	0.264
**Mechanism of injury**					
Other (Machine, animal bite or burn)	6	39	1	1	
Road traffic accident (RTA)	177	205	5.6[2.32–13.56]	5.5[2.27–13.34]	0.000**
Fall accident	68	328	1.3 [0 0.55–3.31]	1.3 [0 0.54–3.27]	0.541
Assault	72	134	3.5[1.41–8.64]	3.4[1.35–8.37]	0.009**
**Comorbidity**					
No	303	682	1	1	
Yes	20	24	1.8[1.02–3.44]	2.2[1.13–4.18]	0.019*

*Significant association at a *P* value of less than 0.05; **Strongly significant association at a *P* value of less than 0.001; *p* value at Hosmer and Lemeshow Test = 0.798, hence the model fits well.

## 4 Discussion

### 4.1 Overall description of the study

In this retrospective health facility cohort study, we measured the incidence, risk factors, and outcomes of traumatic head injury among trauma patients who visited the Yanet Trauma and Surgery Specialized Centre, Ethiopia, in 2023. Over 5 years, 1,029 injured patients were followed for 2,302 person-days. The incidence density rate of head injury was 14.03/100 person-days. The cumulative incidence of head injury among trauma patients was 31.4% [95% CI (29.5–34%)], which was a substantially higher burden. This result was in line with earlier research from Ethiopia (32.1%) (23) and Nigeria (33.8%) (24). On the other hand, our analysis found a higher incidence than a prior research on (12.4%) (25). This discrepancy may have occurred for a variety of reasons, including variations in socioeconomic level, lifestyle characteristics, research design, patient context, and that may raise the risk of head injury.

The most common type of head injury was brain contusion (38.1%), followed by epidural hematoma (33.1%), skull fracture (15.8%), and intracerebral hematoma (13.0%). This finding was comparable with the existing evidence (26–28). Head injuries may occur for a variety of causes. Head injuries can vary based on the type and severity of the impact. For example, a head injury caused by a fall from a height may differ from one caused by a direct blow to the head in a sports-related incident. The force, angle, and speed of the impact can all influence the nature and severity of the injury.

### 4.2 Clinical conditions of the patients

According to this study, 141 (13.75%) of the study respondents presented with poly trauma, and more than half (82 (58.2%) of them) presented with poly trauma. In addition, almost one-third (32.9%, *n* = 339) of the study participants presented with a bone fracture. This finding was consistent with a study conducted in South Africa (29–31). The timeliness and quality of medical care following a head injury can affect its subsequent development and impact. Variations in access to medical facilities, expertise of healthcare providers, and availability of diagnostic tools can contribute to differences in outcomes for individuals with head injuries.

Regarding complications, 15 (5.5%) of the patients developed post traumatic seizures, 13 (1.3%) developed aspiration pneumonia, 7 (0.7%) developed skin contusions, 7 (0.7%) developed extremity paralysis, 5 (0.5%) developed post trauma meningitis, and 4 (0.4%) underwent amputation. These findings are comparable with the documented evidence elsewhere (32–34) indicating that head injury patients experienced other medical and mental health complications during their long-term course and life after injury. The sudden deceleration or direct blows to the head can cause concussions, fractures, contusions, and other types of head injuries.

Our study shows us, the most common investigated modalities used during were X-ray, followed by head computed tomography (CT) and electrocardiogram (ECG). According to existing research, neurosurgical and diagnostic procedures were frequently predicated on identifying shifts in clinical indications and symptoms in the years prior to CT scanning. The patient had an intrusive, hazardous angiography to confirm a diagnosis, with a propensity to delay the procedure until the patient was in critical condition. If digital biomarkers such as hard fall detection are standardized and utilized as a means to notify paramedics to an unresponsive trauma patient, the existing lag in TBI incidence and hospitalization can be eliminated (35).

### 4.3 Risk factors associated with head injury

Considering the factors associated with head injury, socio-demographic characteristics such as rural residence (AOR = 1.6), mechanisms of injury such as RTA (AOR = 5.5) and assault (AOR = 3.4), and comorbidity of chronic disease (AOR = 2.2) were identified as independent predictors of head injury. Thus, the incidence of head injury among rural residents was 60% greater than that among urban residents. This was consistent with the findings of previous studies (20, 36, 37), where traumatic head injuries were more common in rural areas than in urban areas. There are various reasons why rural individuals may have more head injuries than urban ones. Some of the possible reason could be: (a) Rural communities may have restricted access to healthcare services such as hospitals and trauma centers. Delays in seeking early medical treatment after a head injury may result in worse results, (b) Rural populations may be more prone to work in sectors such as agriculture, forestry, or construction, which have a greater risk of head injuries caused by heavy equipment, falls, or blunt force trauma. (c) Rural inhabitants may have to drive greater distances to receive critical services, increasing their chances of being involved in car accidents that result in brain injuries. Poor road conditions and inadequate public transit choices in rural locations may also lead to increased accident rates.

In our study, road traffic accidents and assaults were significantly associated with head injuries. Hence, the prevalence of head injury associated with RTAs and assault (interpersonal violence) was 5.5 and 3.4 times greater, respectively, than that associated with other mechanisms of injury. This finding is also comparable with findings from North Central Ethiopia (20) and Northwest Ethiopia (38). Nevertheless, a research conducted in Australia (1) revealed contrasting results, since the majority of head injuries were attributed to sports and leisure activities rather than road traffic accidents (RTAs) and assault. RTA and assaults may lead to several forms of injury, including ejection from a vehicle, impact with a solid surface, or being hit by a weapon or object. These several mechanisms worsen the intricacy and severity of brain traumas endured.

Furthermore, comorbidities of chronic disease had a significant correlation between these factors and brain injury. Therefore, the incidence of head injury in individuals with comorbidities of chronic illness was 2.2 times higher compared to those without such conditions. The greater incidence of accidents and susceptibility to various consequences among people with chronic conditions may account for this observation. This result was corroborated by studies in Ethiopia (39). Individuals with pre-existing medical conditions, such as epilepsy or neurodegenerative disorders, may be more susceptible to certain types of head injuries or experience different outcomes compared to those without these conditions.

Regarding the clinical condition of the trauma patients, 13.75% (*n* = 141) of the study participants presented with poly trauma, and almost one-third (32.9%) of the study participants presented with bone fractures. This figure was in line with a study performed in the Amhara region, Ethiopia (39). In road traffic incidents, vehicle passengers or pedestrians may not be wearing seat belts, helmets, or other protective gear that may assist lessen the risk of head injuries. Victims of attack may be unable to defend themselves against direct strikes to the head.

### 4.4 Outcomes of head injury

Consecutively, the majority (74.9%) of the study participants had good or improved discharge outcomes. This could be because timely management of head trauma before patients develop secondary brain injury and the use of surgical intervention based on CT scan diagnosis reduce the occurrence of unfavorable outcomes. Furthermore, in the current study, only 16.7% of the study participants had lived for more than 7 days. Therefore, this result implies the need for close follow-up of trauma patients, and the availability of a functional neurosurgery department and strong referral systems in the study area were the main reasons. Hence, these factors have a significant impact on the expected outcome or recovery time for trauma patients. This finding was in line with findings from a study in Nekemte Referral Hospital, Oromia, Ethiopia (40), where the proportion of favorable discharge outcomes was 74.9% (40). Moreover, the current research documented on the trends in mild traumatic brain injury treatment of all types of TBI prior demonstrating the need for improved care specific to mild TBI patients (41). Therefore, the outcomes of head injuries can vary widely depending on the severity of the injury, the specific type of head trauma, the individual’s age and overall health, and the timeliness and quality of medical intervention.

### 4.5 Methodological comparison

The majority of the exiting study were cross-sectional study (42–45) design that measured prevalence of the head injury has lack of temporal relationships. However, the recent study used 5 year observational retrospective cohort study design that helped to outline how data was collected, analyzed, and interpreted. Validity, Reliability, Sample size and representativeness, Validation of measurement tools, Control of confounding variables were considerer to respond research questions. This study had similar methodological issue with the previous study (46–49).

## 5 Strengths and limitations

This study used an adequate and representative sample size to address the research questions. Moreover, data were collected with digital form in kobo with smart phone. However, the limitations of study were retrospective nature of the study design and the lack of data on alcohol consumption and smoking status.

## 6 Conclusion

The incidence of head injury in this study ranged from one to three, which is a significant public health concern in the study setting. In addition, rural residence, RTA, assault, and comorbidity of chronic disease were identified as independent predictors of head injury. Therefore, policy makers and partners should focus on tailored strategies such as wearing protecting helmets, practice safe driving with seatbelt while in a vehicle, maintain a safe environment, avoid risky behaviors such as excessive drinking/drug use or smoking, consult a healthcare provider for screening and early treatment of comorbidity are very essential to mitigate the risk of head injury.

## Data Availability

The original contributions presented in the study are included in the article/supplementary material, further inquiries can be directed to the corresponding author.
